# A two-step screen identifies a small molecule that disrupts membrane voltage and is effective against growing and persister gram-negative bacteria

**DOI:** 10.1128/spectrum.03217-25

**Published:** 2025-12-31

**Authors:** Ciara K. Asamoto, Calvin A. Ewing, Christian T. Meyer, Samual C. Allgood, Matthew J. G. Eldridge, Donald Evans, Toni A. Nagy, Grace L. Christensen, Amy L. Crooks, Daqing Jiang, Sophie Helaine, Corrella S. Detweiler

**Affiliations:** 1 Department of Molecular, Cell, and Developmental Biology, University of Colorado Boulder 1877, Boulder, Colorado, USA; 2 Department of Microbiology, Harvard University 1812, Cambridge, Massachusetts, USA; JMI Laboratories, North Liberty, Iowa, USA

**Keywords:** antibiotic, anti-infective, cell envelope, macrophage, membrane, *Salmonella Typhimurium*

## Abstract

New antibiotics effective against gram-negative bacteria are difficult to discover because many small molecules are excluded by the cell envelope. There is a growing consensus that the proton motive force (PMF) represents a key target for the development of antimicrobial agents. Here, we report data that both support that consensus and demonstrate the utility of host-based screens in discovering novel antibiotics. Previous in-host screens of small-molecule libraries have identified compounds that inhibit *Salmonella enterica* serovar Typhimurium (*S*. Typhimurium) survival within macrophages but lack potency in standard broth alone. This suggests that infection conditions may facilitate penetration of small molecules due to host-mediated damage to the bacterial cell envelope, confirming the potential for streamlined host-based screens to identify new antibiotics. Here, we screened a library of drug-like compounds in broth with an outer membrane-permeabilizing agent. Eighty compounds were identified and selected for inhibiting bacterial proliferation in macrophages. CB1.11, a mildly lipophilic compound predicted to embed in the inner membrane, was particularly effective. We found that CB1.11 targets the inner membrane to weaken the PMF, disrupt voltage-dependent protein localization, and enhance the ability of the host cell to clear the pathogen. Additionally, CB1.11 reduces persister cell survival in broth and macrophages and demonstrates antibacterial activity in gram-negative and gram-positive bacteria. These data provide evidence that the PMF is a critical target for antimicrobial development and demonstrate the utility of using streamlined host-based screens to discover novel antibiotics.

**IMPORTANCE:**

Finding new antimicrobials for gram-negative bacteria is challenging because their outer membrane is difficult to penetrate. Standard laboratory screens often miss promising candidates since they do not reflect real infection conditions, where the host environment weakens the gram-negative outer membrane and allows compounds better access to their targets. In this study, we developed a simple two-step screening method to enhance antimicrobial discovery in host-based screens. First, we tested compounds in broth that mimics host conditions to find those likely to work during infection. We then screened this smaller group in infected immune cells to identify molecules that limit pathogen survival. We identified a compound that disrupts the bacterial inner membrane and energy production, helping host cells clear the infection. The compound also effective against gram-positive bacteria and persister cells. These findings show the potential of developing antimicrobials that target bacterial energy production and the benefits of using host-based screening platforms.

## INTRODUCTION

There is an urgent need for new approaches to meet the growing crisis of bacterial antibiotic resistance. Infections caused by gram-negative bacteria are particularly difficult to treat due to the densely packed outer membrane that excludes many small molecules from the bacterial cell (1). Additionally, compounds that do manage to traverse the outer membrane are met with Resistance-Nodulation-Cell-Division (RND) efflux pumps that can export small molecules, including diverse classes of antibiotics, from the periplasm across the outer membrane (2). Complicating the treatment landscape further is the presence of persister cells, a genetically identical yet phenotypically distinct subpopulation of cells that are nongrowing and can survive in the presence of antibiotic treatment (3, 4). Persisters are suspected to contribute to infection relapse as they are able to regrow once treatment has ceased (5, 6). Consequently, the development of antibiotics that are effective against gram-negative pathogens across both growth and persister states is both highly desirable and difficult to achieve.

Traditional screens for antibacterial compounds have been carried out in nutrient-rich broth (7, 8). These are not conditions bacteria typically encounter within a host and, consequently, compounds that may block bacterial growth under laboratory conditions may fail to impact bacteria during infection of host cells or animals. Host-cell-based screens for antimicrobial compounds have been developed and have identified molecules that target either pathogen or host factors with minimal host toxicity (9–12). For example, the Screen for Anti-infectives using Fluorescence microscopy of IntracellulaR Enterobacteria (SAFIRE) platform is an in-macrophage high-content confocal fluorescence-based assay that identifies compounds that reduce survival and proliferation of the gram-negative human pathogen *Salmonella enterica* serovar Typhimurium (*S*. Typhimurium) while counter-screening for host-cell integrity (12, 13). In this assay, the bacterial load per macrophage is quantified with a bacterial GFP-reporter, and macrophage viability is monitored based on the morphology and staining with MitoTracker Red, an indicator of mitochondrial membrane voltage.

Screens of compound libraries with the SAFIRE platform have uncovered previously overlooked compounds that have low antibiotic activity under laboratory conditions but are effective against bacteria in host cells (14). For example, clofazimine, an antibiotic used against Mycobacterium, lacks activity against gram-negative bacteria in broth but is antibacterial in the context of infection (14). These data highlight the potential that host-based screens have for revealing novel anti-infectives that standard broth media assays fail to identify, presumably because these assays do not represent infection conditions.

Host-based screens are resource-intensive, which limits their utility in screening large libraries. By leveraging prior observations on compounds identified using the SAFIRE assay, we developed a two-step screening strategy that reduces the size and complexity of this host cell-based screen. Many effective compounds only inhibit bacterial growth in standard broth under conditions that reduce the barrier effect of the cell envelope. These data suggest that the compounds may be effective in macrophages where they are potentiated by native host defenses that weaken the bacterial cell envelope barrier by either increasing the outer membrane permeability or decreasing RND efflux pump activity (12, 14–16). Therefore, prior to conducting a screen using the SAFIRE assay, we pre-screened for compounds that, in combination with a cationic antimicrobial peptide (CAMP), prevent the growth of gram-negative bacteria in broth. Positively charged CAMPs disrupt the negatively charged lipopolysaccharide (LPS) layer in the outer membrane, leaving the bacteria vulnerable to antimicrobials (17). Moreover, CAMPs accumulate within macrophage phagosomes that harbor bacterial pathogens (18). We therefore reasoned that CAMP permeabilization of the outer membrane would be analogous to the effect of host soluble innate immunity on bacteria during infection, enabling for a subset of compounds that are more likely to be antibacterial during infection to be enriched in a broth assay. We applied this two-step screening strategy to a small-molecule chemical diversity library of approximately 17,000 compounds from the ChemBridge (CB) CombiSet Library. We then examined how the hit compound CB1.11 inhibits *S*. Typhimurium during infection-like conditions using a combination of broth assays and molecular dynamics (MD) simulations. Finally, we examined whether CB1.11 impacts *S*. Typhimurium persister cells in broth and in macrophages.

## RESULTS

### A two-step screen reveals small molecules that prevent *S*. Typhimurium proliferation in macrophages

**Fig 1:**
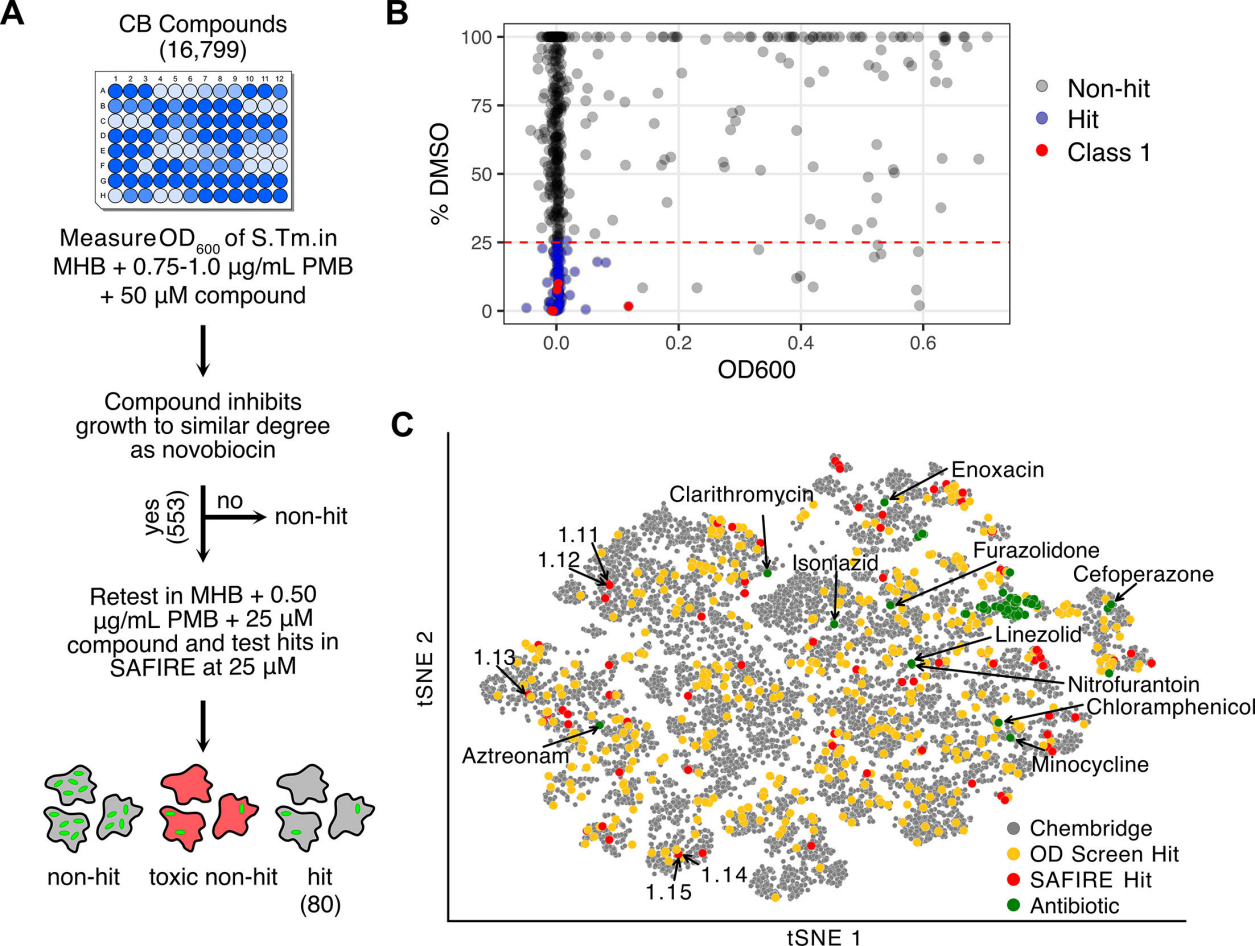
Two-step screening protocol identified hit compounds from across a broad landscape of the CombiSet library. (**A**) The first screening step for compounds that inhibit growth in the presence of PMB identified 553 hits that were screened in the second set i) at 25 µM against *S*. Typhimurium in MHB supplemented with 0.5 µg/mL PMB (not shown) and ii) for inhibition of bacterial accumulation in macrophages at 25 µM in a SAFIRE assay. Compounds that inhibited bacterial growth to a similar degree as novobiocin controls reduced GFP signals within macrophages by >75% relative to DMSO and did not show toxicity retained hit status. (**B**) Hit compound activity in the SAFIRE assay (% DMSO) vs growth inhibitory activity with 0.5 µg/mL PMB and 25 µM compound (OD_600_). Nonhit compounds are colored gray, hit compounds are colored blue, and Class 1 compounds are colored red. The % DMSO values greater than 100% are plotted as 100%. The dashed red line indicates the cutoff for the SAFIRE assay (GFP signal reduced by >75% relative to DMSO). (**C**) t-Stochastic Neighborhood Embedding (tSNE) clustering of the 16,799 library compounds and standard antibiotics. Class 1 compounds and antibiotics that clustered away from the main antibiotic cluster are annotated. Distance calculated using Tanimoto similarity. See Fig. S2 for tSNE clustering of antibiotics independent of the library and Table S2 and S3 for full list of antibiotics, classes, and chemical properties.

The ChemBridge Combi-PremiumSet arrayed library of drug-like small molecules was selected for screening based on its broad chemical space representation and high diversity. In the first screening step, we monitored *S*. Typhimurium growth in the presence of compound and the cAMP polymyxin B (PMB) over 18 h. A bacterial culture was incubated with 16,799 ChemBridge compounds at 50 µM in Muller-Hinton broth (MHB) supplemented with PMB in a 96-well format (Fig. 1A; Table S1). Control wells were treated with DMSO, the library solvent, with and without PMB to provide measures of maximum growth. Across all plates, the control with PMB wells grew to approximately 80% of the wells that received DMSO alone. As expected, most compounds had little to no effect on *S*. Typhimurium growth in the presence of PMB. Additional control wells were treated with the antibiotic novobiocin, an LPS-impermeant small molecule, which is potentiated by PMB (19). We used PMB at concentrations high enough (0.75 or 1.0 µg/mL) to permeabilize the *S*. Typhimurium outer membrane, but low enough to leave the inner membrane intact, as determined empirically (14). We identified 553 hit compounds (2.8% of the total library) that acted as growth inhibitors in the presence of PMB.

Each of the 553 compounds was re-tested for growth inhibition of *S*. Typhimurium in MHB and examined for efficacy in the SAFIRE assay (Fig. 1B; Table S2). In the growth-inhibition assay, compounds were tested at a concentration of 25 µM and with a reduced concentration of PMB (0.5 µg/mL) to increase stringency (i.e*.*, the compounds must maintain efficacy with less PMB present). In the SAFIRE assay, hit compounds were tested at 25 µM in infected RAW 264.7 mouse macrophage-like cells using a *S*. Typhimurium strain with a chromosomal *sifB::gfp* reporter that expresses GFP only when *S*. Typhimurium is within macrophages (12). After 15.5 h of compound treatment, cells were stained with the vital dye MitoTracker Red, fixed, and stained with DAPI. Confocal images were masked and evaluated for GFP (bacterial) signals per macrophage. Eighty compounds were found to reduce GFP signals by >75% relative to DMSO in SAFIRE and reduced *in vitro* growth of *S*. Typhimurium in MHB with PMB by >90% relative to DMSO retained their “hit” status (blue dots, Fig. 1B). Together, the 553 hit compounds from the first step of the screen comprise 3.3% of the library of the compounds tested. Of these, 14.5% were antibacterial in the SAFIRE assay.

### Analysis of hit compounds

To establish how chemical characteristics of the library compounds and the hits from the two-step screen compare, we visualized the chemical diversity of the hits using tSNE (Fig. 1C). Overall, the 553 hits from the first screening step (yellow dots, Fig. 1C) were broadly distributed across the chemical space of the library (gray dots, Fig. 1C), indicating that chemically diverse small molecules inhibit bacterial growth in the presence of PMB. A similar dispersion was observed for the 80 hit compounds that were retested in the presence of PMB and were also active in the SAFIRE assay (red dots, Fig. 1C), confirming that these chemically diverse compounds are both bioavailable in infected cells and possess antibiotic activity. To determine how our hits are compared with existing clinical antibiotics, 138 antibiotics spanning 21 distinct structural classes were embedded in the same tSNE manifold (green dots, Fig. 1C). In comparison to the SAFIRE hits and the library as a whole, the antibiotics clustered together, indicating limited chemical diversity in current clinical antibiotics. As a control, we embedded the antibiotics independently of the library and observed that they clustered according to their canonical annotation (Fig. S1), demonstrating that the clustering algorithm correctly groups antibiotic classes. In summary, the t-SNE analysis suggests that compounds with antibacterial activity in macrophages encompass diverse chemical space not represented by current clinical antibiotics.

To determine whether particular chemical properties are strong predictors of hit compounds, we performed a principal component analysis (PCA) on different features of the compounds in the screen (Fig. S2A). Increased lipophilicity (i.e., logP) was overrepresented in the hit compounds compared to the distribution of the library. We additionally evaluated logP values from hit compounds that were identified in two previous SAFIRE screens (12, 14). We noted a modest increase in logP values in the hit compounds relative to the parent libraries (an increase of between 0.5 and 1.0), suggesting that SAFIRE hits are enriched for lipophilic compounds (Fig. S2B). We conclude that while hits that derive from an in-macrophage infection screening platform tend to be lipophilic, neither increased lipophilicity nor any other chemical attribute examined is predictive of a hit compound (Fig. S2A). The 80 hits were then manually grouped into 12 classes consisting of 2–14 compounds each based on similarities in their chemical structures (Table S2).

### Class 1 compounds show potential as antimicrobials

**Fig 2:**
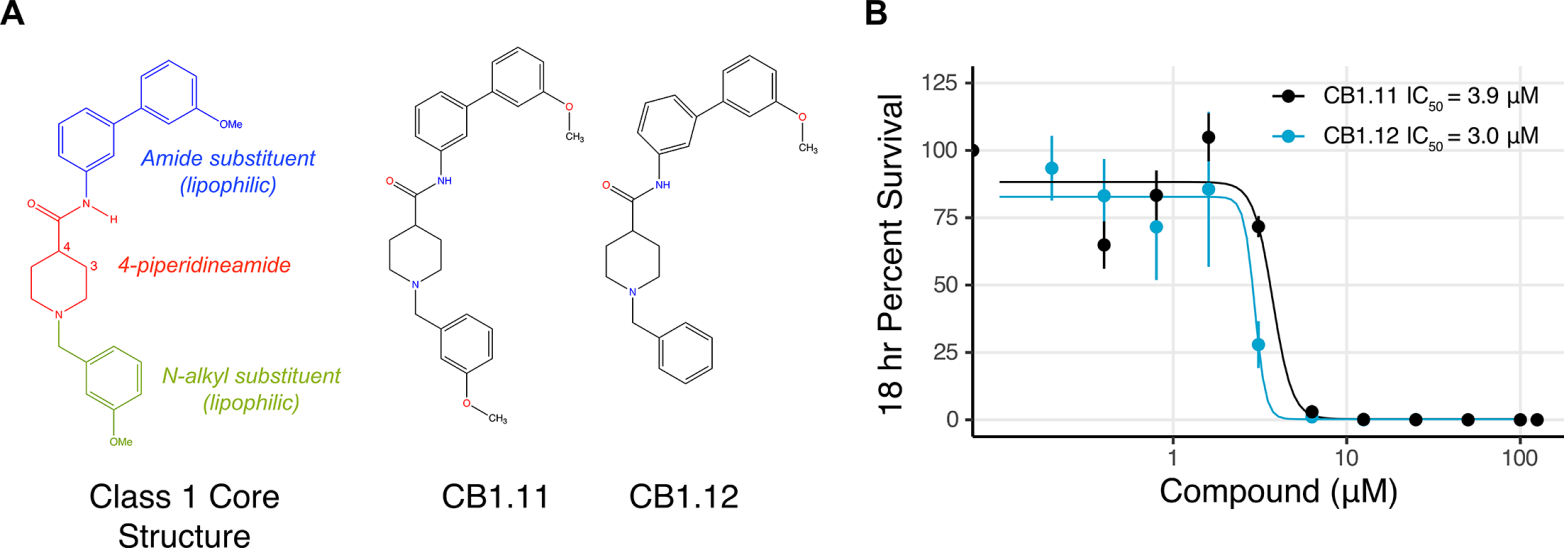
Class 1 compound structures and activity against intracellular *S*. Typhimurium. (**A**) Core structure of the Class 1 compounds and chemical structures of CB1.11 and CB1.12. Class 1 compounds share a piperidineamide core with amide and N-alkyl substituents. (**B**) Dose-response curves for compounds CB1.11 and CB1.12 against *S*. Typhimurium within RAW 264.7 macrophages. After 18 h of infection, macrophages were lysed and bacterial survival estimated via CFU counts. Data were normalized to DMSO control conditions for each replicate. The key displays IC_50_ values. Mean of three biological replicates with SEM.

The Class 1 compounds were selected for study because two of them, CB1.11 and CB1.12, strongly inhibited *S*. Typhimurium growth in the SAFIRE assay and in MHB with PMB (Table S1 ). Class 1 consists of three compounds characterized by a 3- or 4-piperidineamide core with N-alkyl and amide substituents (Fig. 2A). To establish whether the decrease in GFP signals in the SAFIRE assay specifically reflected bacterial death and/or growth inhibition rather than compound interference with GFP expression, we monitored bacterial survival in macrophages by enumeration of colony-forming units (CFU). Infected RAW cells were treated with a range of compound concentrations, lysed, and then plated for bacterial enumeration via CFU (Fig. 2B). The concentration required to reduce the number of CFUs by 50% (IC_50_) was calculated for CB1.11 and CB1.12, with IC_50_ values of 3.9 and 3.0 µM, respectively. However, the MitoTracker Red staining from the SAFIRE assay suggested higher signal from CB1.12, indicating that this compound could cause more metabolic stress to the macrophages than CB1.11 (Fig. S3A). CB1.11 was also less toxic than CB1.12, as determined by a lactate dehydrogenase release assay, with a half-maximal cell cytotoxicity (CC_50_) of 30 µM, compared to 19.7 µM for CB1.12 (Fig. S3B and C). The selectivity index (CC_50_/IC_50_) for CB1.11 was computed to be 7.8 (Fig. S3C). Consequently, CB1.11 was selected as a representative from the Class 1 compounds for further testing.

### Weakened cell envelope integrity enables CB1.11 to inhibit bacterial growth

**Fig 3:**
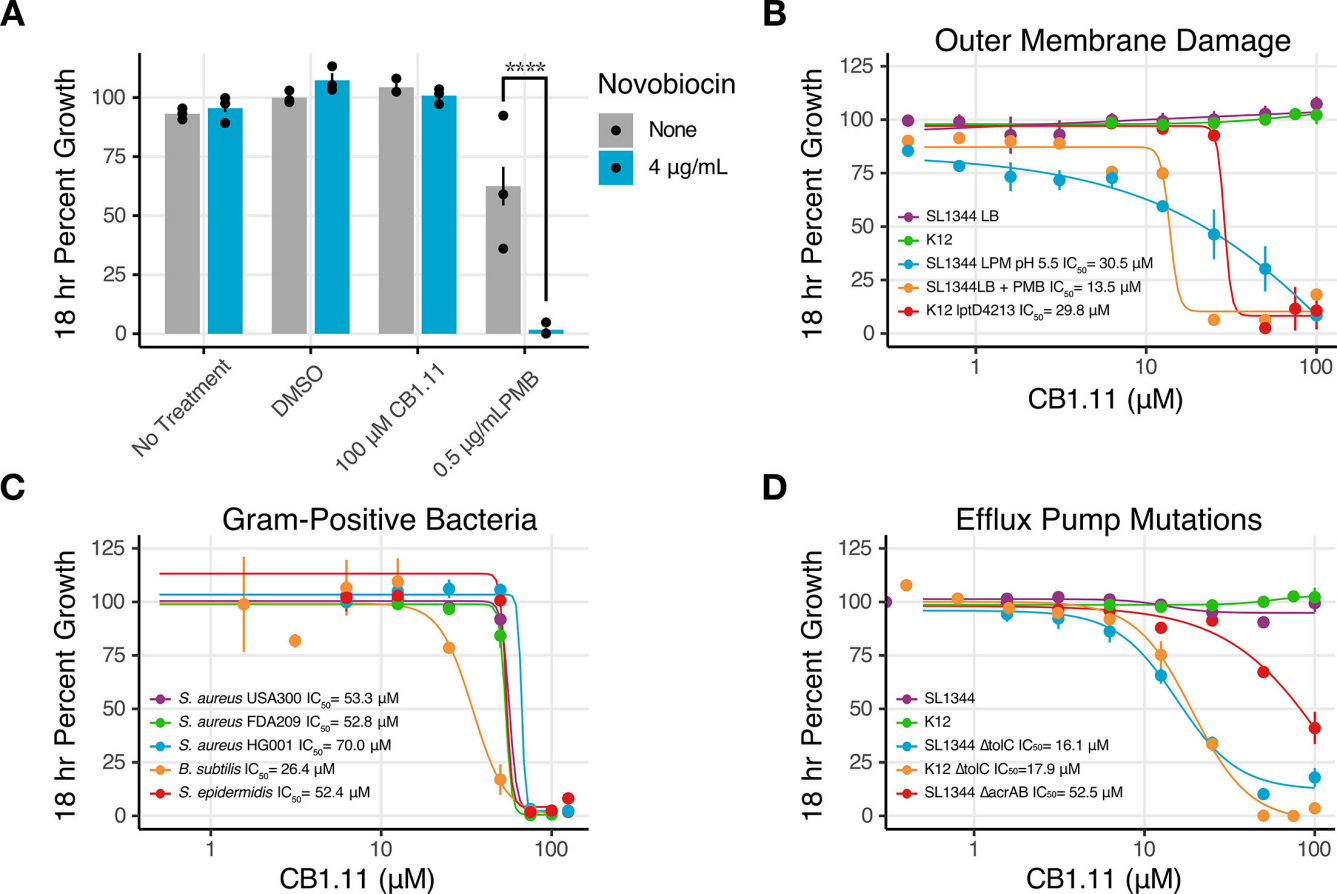
The compound CB1.11 does not appear to permeabilize the outer membrane and inhibits growth under conditions that either compromise the gram-negative cell envelope or in gram-positive bacteria, which lack an outer membrane. (**A**) SL1344 growth at 18 h in LB with or without novobiocin, as indicated. Samples included no treatment, DMSO, 100 µM CB1.11, or 0.5 µg/mL PMB. Error bars represent the mean ± SEM of three biological replicates. Statistical analysis by two-way ANOVA and a Tukey’s post test, **** adjusted *P* < 0.0001. Dose-response curves of CB1.11 against (**B**) *S*. Typhimurium SL1344 grown in LB, LB supplemented with 1 µg/mL PMB, or LPM at pH 5.5, or *E. coli* K12 grown in LB, or the barrier mutant *E. coli* K12 *lptD4213* grown in LB. (**C**) Gram-positive bacteria grown in TSB, and (**D**) SL1344 and K12 *∆tolC* mutant strains and SL1344 *∆acrAB* mutant strain grown in LB. (**B–D**) Growth was monitored by OD_600_ over 18 h. Data were normalized to DMSO control conditions for each replicate. Calculable IC_50_ values are displayed. Mean and SEM of three biological replicates. K12 data presented in B and D are the same experimental data.

Previous compounds identified using the SAFIRE screen target the bacterial inner membrane without permeabilizing the outer membrane (15, 16). Thus, we tested whether CB1.11 could enable the antibiotic novobiocin to cross the outer membrane to inhibit growth (20). As anticipated, novobiocin treatment alone had no impact on *S*. Typhimurium growth in LB, in which the outer membrane remains fully intact (Fig. 3A). In the presence of PMB alone, *S*. Typhimurium was inhibited to ~60% of maximum growth relative to DMSO controls, and PMB with the addition of novobiocin completely inhibited growth. In contrast, the addition of novobiocin to lysogeny broth (LB) with 100 µM of CB1.11 did not inhibit growth, demonstrating that CB1.11 does not permeabilize the outer membrane to other compounds.

We next established whether conditions that damage outer membranes or compromise bacterial efflux pumps, integral components of the cell envelope, are necessary to enable growth inhibition by CB1.11. We inoculated *S*. Typhimurium or *E. coli* into LB with concentrations ranging up to 100 µM of the compound, and no growth inhibition was observed after 18 h (green and purple lines, Fig. 3B). As expected, PMB exposure (1 µg/mL) strongly potentiated the growth inhibitory activity of CB1.11, with an IC_50_ value of 13.5 µM (orange line, Fig. 3B). Two other conditions previously demonstrated to weaken outer membrane integrity were also examined: a low-magnesium, low-phosphate, and low-pH medium developed to mimic the phagosome environment and destabilize LPS (LPM 5.5) and *E. coli* K12 with an *lptD4213* mutation that increases the permeability of the outer membrane (21–23). Both the LPM 5.5 medium and the *lptD4213* mutant strain also enabled CB1.11 to inhibit growth, with IC_50_ values of 30.5 and 29.8 µM, respectively, suggesting that CB1.11 is potentiated by a weakened outer membrane barrier (blue and red lines, Fig. 3A). We also tested CB1.11 against representative gram-positive bacteria because they lack an outer membrane. *Staphylococcus aureus, S. epidermidis,* and *Bacillus subtilis* growth was modestly inhibited by CB1.11 in broth culture in the absence of membrane-permeabilizing conditions. It was noted that the Staphylococcal strains were less sensitive than *B. subtilis* (IC_50_ between 52.8 and 70.0 µM vs 26.4 µM, Fig. 3C). These data support the idea that a target(s) of CB1.11 is conserved across gram-negative and gram-positive bacteria and that CB1.11 has low permeability across the gram-negative outer membrane.

Since permeabilization of the outer membrane in broth culture sensitized bacteria to CB1.11 but did not improve antibacterial activity to the degree observed in macrophages (Fig. 2C), we hypothesized that CB1.11 was a possible substrate of RND efflux pumps. To test this idea, we cultured *S*. Typhimurium and *E. coli ∆tolC* mutant strains in LB in the presence of CB1.11 and monitored growth. TolC is an outer membrane channel protein that pairs with all five *S*. Typhimurium and *E. coli* RND efflux pumps; in strains lacking *tolC*, RND efflux is severely compromised (24). *S*. Typhimurium and *E. coli ∆tolC* strains were both sensitive to CB1.11 in LB, with IC_50_ values of 16.1 and 17.9 µM, respectively (Fig. 3D). Since the *∆tolC* strains were tested under conditions that did not compromise outer membrane permeability, these data suggest that CB1.11 can pass through the outer membrane unaided and that the compound is, in addition, a substrate for efflux. However, it has been suggested that deletion of *tolC* may slightly increase the outer membrane permeability (25, 26). Thus, we also tested CB1.11 for growth inhibition of a *∆acrAB* mutant as this locus encodes the periplasmic and inner membrane subunits of the major RND efflux pump required for *S*. Typhimurium pathogenesis, AcrAB-TolC. Compared to the wild-type SL1344 strain, the ∆*acrAB* mutant was sensitive to CB1.11, with an IC_50_ of 52.5 µM. As expected, the ∆*acrAB* strain was less sensitive to CB1.11 than the *∆tolC* strain, which had an IC_50_ of 16.1 µM. This observation is consistent with the role TolC plays as the outer membrane channel for multiple efflux pumps as well as its possible contribution to outer membrane integrity. Ultimately, the data suggest that CB1.11 has the ability to traverse an intact outer membrane but is also captured and exported by RND efflux pumps. Thus, growth under conditions that compromise efflux pump activity and/or outer membrane integrity potentiates the growth-inhibitory activity of CB1.11.

### Antimicrobial activity of CB1.11 correlates with the predicted ability to insert into bacterial membranes

**Fig 4:**
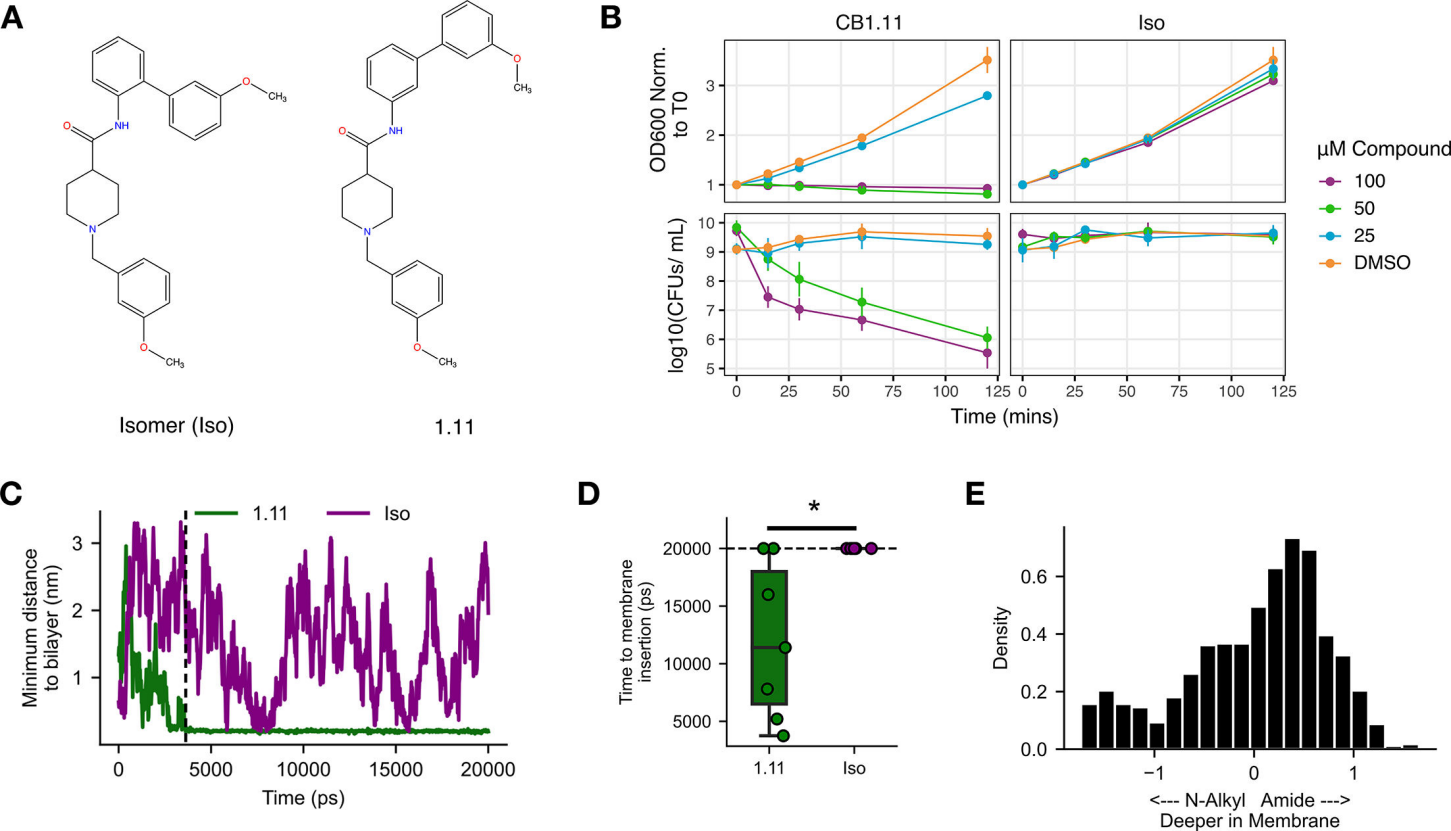
The Iso of CB1.11 is unable to inhibit bacterial growth and is predicted to inefficiently embed into bacterial inner membranes. (**A**) Structure comparison of CB1.11 and the CB1.11 Iso, which contains an ortho- rather than a meta-substituted amide bond linking the benzene functional group. (**B**) Growth of mid-log phase *S*. Typhimurium ∆*tolC* in LB with three doses of CB1.11 or Iso, as measured by OD_600_. Data are normalized to OD_600_ at time 0 (top). Viability of mid-log phase *S*. Typhimurium ∆*tolC* in LB after treatment with CB1.11 or Iso, as measured by the geometric viability assay (bottom). Mean and SEM of three biological replicates. (**C**) Predicted distance of CB1.11 and Iso from a bacterial specific lipid bilayer based on MD simulations. Compounds were solvated above the membrane prior to system equilibration. The simulation was run for 20 nanoseconds (ns). Minimum distances between the center of mass for each compound and each membrane lipid are plotted. The dashed line indicates the time where CB1.11 is inserted into the membrane. (**D**) Time to membrane insertion for CB1.11 and Iso across *n* = 7 and *n* = 6 simulations, respectively. Insertion was defined as persistent proximity of < 2.5 Å. Statistics calculated using two-sided *t*-test (*P < 0.05). The dotted line indicates the time limit of simulations. (**E**) Membrane orientation of CB1.11. The X-axis is the difference in the z-position between N-alkyl and amide ends of CB1.11. The Y-axis shows the probability of that orientation post-membrane insertion across all simulations.

A custom-synthesized batch of CB1.11 was found to lack growth-inhibitory activity. Assessment of this batch with mass spectrometry and UV spectral analyses revealed that the molecule had an identical chemical composition to CB1.11 but contained an ortho- rather than a meta-substituted amide bond linking the benzene functional group (Fig. 4A). This subtle difference was apparently enough to render the isomer (Iso) ineffective against the *S*. Typhimurium *∆tolC* mutant strain in growth and survival assays (Fig. 4B), providing a unique opportunity for investigation of CB1.11 activity.

To explain the discrepancy between CB1.11 and Iso activity, we examined their distinguishing chemical features. Most notable was the difference in the calculated partition coefficient for CB1.11 and Iso (logP = 4.55 and 3.73, respectively, Daylight ToolKit’s CLOGP calculation method). We reasoned that if CB1.11 antibacterial activity depended on interactions with the inner membrane, the decrease in the hydrophobicity of Iso could contribute to the difference in activity. To explore this possibility, we ran MD simulations, with each compound solvated above a lipid bilayer with a lipid composition consistent with gram-negative inner membranes (27, 28). Over the course of a 20 ns simulation, CB1.11 embedded into the membrane while Iso remained solvated despite several close encounters (<2.5 Å) with the bilayer (Fig. 4C; Fig. S4A and B). In replicate simulations with different random variable initialization, Iso was never found to embed in the membrane over the 20 ns of simulation time, whereas CB1.11 embedded in the membrane in approximately 70% of simulations (Fig. 4D). Post-insertion, the amide group of CB1.11, where Iso has the ortho-substituted moiety, tended to embed deeper into the membrane relative to the N-alkyl group across all simulations (Fig. 4E). We also found that CB1.11 had no apparent preference for any particular lipid and that the top three lipid groups it associated with, plasma membrane phosphatidylethanolamine (PMPE), plasma membrane phosphatidylglycerol (PMPG), and 1-palmitoyl-2-oleoylphosphatidylethanolamine (POPE), reflect the main composition of the inner membrane in gram-negative bacteria (Fig. S4C). In conclusion, the antibacterial properties of CB1.11 depend on the ability of the compound to interact with bacterial cell membranes.

### CB1.11 affects *S*. Typhimurium by disrupting membrane voltage without overt membrane lysis

**Fig 5:**
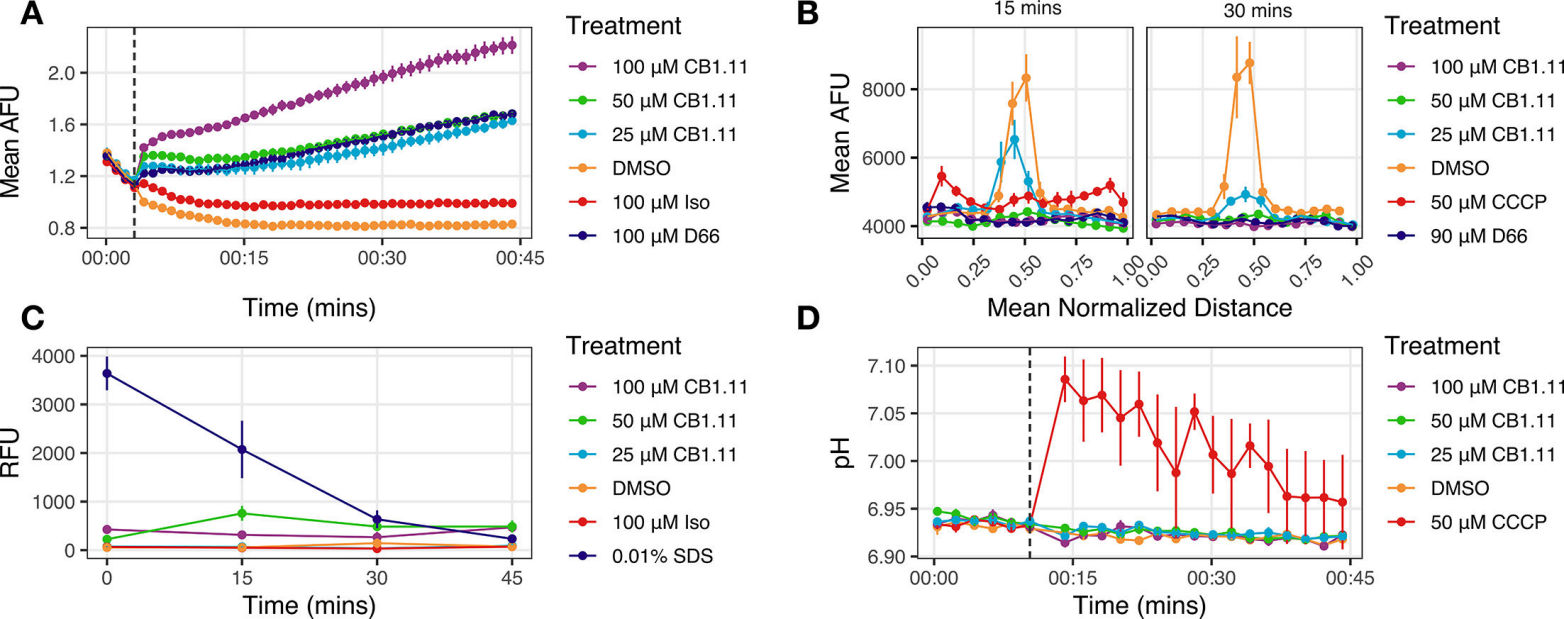
CB1.11 subtly disrupts membrane voltage without permeabilizing the inner membrane nor disrupting the pH gradient. (**A**) Membrane polarity in response to CB1.11 was measured by DiSC_3_(5) fluorescence in *S*. Typhimurium *∆tolC* grown in LPM pH 7.0. The dashed line indicates when compound was added. Mean ± SEM of three biological replicates. (**B**) Response of *S*. Typhimurium *∆tolC* grown in LB after treatment with CB1.11, as established by localization of GFP-FtsZ fluorescence intensity to the septum. Data are line scans across 20 cells for which bacterial length was normalized to 1 and then placed into 15 bins for size; each bin is a data point. Means and SEM were calculated based on the observations per each distance bin. Three biological replicates with 20 bacterial cells per condition were performed. (**C**) Inner membrane integrity of *S*. Typhimurium *∆tolC* grown in LPM pH 7.0 was tracked with propidium iodide (PI) fluorescence in the presence of DMSO, 0.01% SDS, CB1.11, and 100 µM Iso. Increases in PI fluorescence indicate a compromised inner membrane. Mean ± SEM of three biological replicates. (**D**) Response of cytosolic pH of *S*. Typhimurium ∆*tolC* in the presence of CB1.11 in LPM pH 7.0, as measured with 2’,7′-Bis(2-Carboxyethyl)−5-(and-6)-Carboxyfluorescein, Acetoxymethyl Ester (BCECF, AM). The protonophore CCCP was used as a positive control for intracellular pH disruption. Dashed lines indicated when BCECF was added. Mean ± SEM of three biological replicates.

Given the lipophilicity of CB1.11 and the potential for this compound to embed into bacterial membranes, we hypothesized that CB1.11 may target and damage the inner membrane. Inner membrane integrity is key for cellular homeostasis, and, in particular, for maintaining proton motive force (PMF). PMF is dependent upon gradients of voltage and of pH across the inner membrane. We therefore hypothesized that CB1.11 may disrupt one or both of these gradients. To determine whether membrane voltage, also referred to as membrane potential, is affected by CB1.11, we monitored inner membrane polarization of the *S*. Typhimurium *∆tolC* strain using the dye 3,3'-dipropylthiadicarbocyanine iodide (DiSC_3_(5)). DiSC_3_(5) is incorporated into polarized membranes where its fluorescence is quenched. If a compound disrupts the inner membrane and membrane voltage cannot be sustained, the dye is released, causing an increase in fluorescence signals. The *∆tolC* strain was selected so that we could study CB1.11 effects amplified in batch culture upon accumulation of the compound within the cell. These experiments were conducted in LPM at neutral pH because low pH interfered with DiSC_3_(5) fluorescence. In LPM pH 7, exposure to Iso (100 µM) slightly increased fluorescence above that of DMSO (Fig. 5A). In contrast, exposure to CB1.11 strongly increased fluorescence in a dose-dependent manner and was more potent than the positive control compound D66 (15). We conclude that CB1.11 disrupts the voltage gradient across the inner membrane.

We next investigated whether CB1.11 causes voltage disruption at the single-cell level with an *S*. Typhimurium strain expressing a *ftsZ::gfp* reporter. FtsZ requires membrane potential to assemble into a ring at the septum during cell division (29). In previous work, we quantified the number of cells with FtsZ rings that were intact versus diffuse as a proxy for membrane voltage disruption (30). Here, we quantified FtsZ intensity across the length of bacteria with line scans of individual cells. We examined the distribution of GFP signals in cells treated with DMSO, CCCP, D66, or CB1.11. In the DMSO control, we observed a spike in the fluorescence intensity in the midsection of the cells, consistent with localization of FtsZ at the septum (orange line, Fig. 5B). As expected, the relatively flat line-scan profile for cells exposed to the protonophore CCCP, which causes PMF collapse, was consistent with FtsZ delocalization from the septum. After 15 min, we observed a modest peak in the fluorescence intensity in cells exposed to CB1.11 (25 μM) treatment and a flat line scan at higher concentrations, indicating that CB1.11 disrupted bacterial membrane voltage in a dose-dependent manner. After 30 min, cells exposed to 25 µM of CB1.11 had further reduced FtsZ fluorescence at the septum. Overall, these data show that treatment with CB1.11 can cause the dissolution of the FtsZ ring that is required for cell division, indicating that it actively disrupts membrane potential and, in consequence, cell division (Fig. 5B).

Given that CB1.11 disrupts inner membrane voltage, we hypothesized that it could affect membrane barrier function and enable a molecule as large as PI, 668 g/mol to enter the cell. PI intercalates into DNA and fluoresces, such that increases in fluorescence correlate with increased inner membrane permeability. As expected, DMSO-treated cells accumulated almost no fluorescence over 45 min, whereas sodium dodecyl sulfate (SDS)-treated cells had the highest amount of fluorescence immediately after treatment (Fig. 5C). Using an ANOVA and a Dunnett’s post-test with DMSO as the control group, we found that CB1.11-treated cells did not accumulate significant levels of PI over 45 min at any concentration, with the only statistically significant accumulation of PI occurring in the SDS-treated cells during the first 30 min. These data suggest that while CB1.11 rapidly disrupts membrane voltage at concentrations as low as 25 µM, membrane barrier function remains intact.

To establish whether the pH gradient component of PMF is affected by CB1.11, we used the cytosolic pH indicator BCECF, AM, and LPM at pH 7.0 to facilitate CB1.11 passage through the outer membrane in the *S*. Typhimurium *∆tolC* strain. Immediately after the addition of CCCP, the cytosolic pH increased from 6.93 ± 0.0 to 7.08 ± 0.05 and slowly recovered over the following 35 min. In contrast, there was no significant difference in pH between DMSO and CB1.11 treated bacteria (Fig. 5D). We conclude that CB1.11 exposure does not impact cytosolic pH and so does not likely enable proton movement across the inner membrane. Overall, it appears that exposure to CB1.11 depletes PMF specifically by disrupting the voltage gradient and not the proton gradient and that CB1.11 exposure does not enable larger molecules to freely pass through the inner membrane.

### CB1.11 reduces persister populations

**Fig 6:**
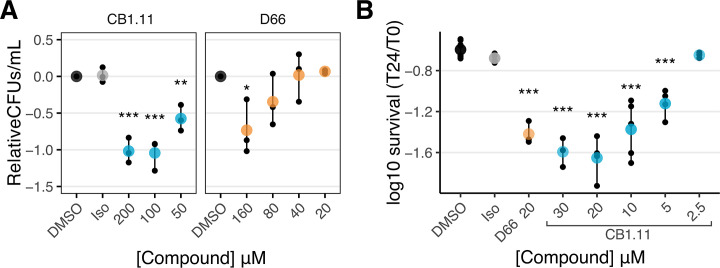
Small molecules that disrupt membrane voltage reduce persister populations in broth and macrophages. (**A**) Broth experiments in LPM pH 7 with *S*. Typhimurium SL1344 exposed to CB1.11 and D66. After 2 h of outgrowth, 25 µg/mL cefotaxime was added to the cultures, and then 2 h later, a dilution series of CB1.11 or D66 was added. Error bars report the maximum and minimum values of the biological replicates, which are plotted as black points. The average of all the biological replicates is plotted as a larger and semi-transparent point. At 6 h, the means of each treatment were compared using an ANOVA followed by a Dunnett’s post test. Each biological replicate (*n* = 3) was normalized to its respective DMSO control to account for variability in persister population size. *P* of < 0.05, 0.005, and 0.0005 denoted by *, **, and ***, respectively. (**B**) Log_10_ survival of SL1344 persisters after 24 h of immortalized bone marrow-derived macrophage (iBMDM) infection. iBMDMs were infected with *S*. Typhimurium at an MOI of 30 for 30 min and then treated with 100 µg/mL cefotaxime and treated with either CB1.11 or D66. DMSO and Iso served as negative controls. After 24 h, iBMDMs were lysed, and then CFUs were enumerated the next day. Error bars report the maximum and minimum values of the biological replicates plotted as black points. The average of all the biological replicates is plotted as a larger and semi-transparent point. A total of three to six biological replicates were completed for each treatment. Outliers were identified using a Grubbs test and were removed before statistical analyses were done. The means of each treatment were compared to the DMSO control using a Dunnett’s test. *P* < 0.05, 0.005, and 0.0005 denoted by *, **, and ***, respectively.

Maintaining inner membrane voltage is a critical aspect of bacterial homeostasis in both actively dividing cells and persister populations. Intramacrophage *S*. Typhimurium persisters are metabolically active and require energy to maintain their efflux pumps and DNA repair machinery despite not actively proliferating (5). Given that CB1.11 appears to disrupt inner membrane voltage, impeding energy-generating processes, we tested whether CB1.11 and D66, another membrane voltage-disrupting small molecule (15), reduces the survival of *S*. Typhimurium persisters in batch culture. To select for the persister cell population, *S*. Typhimurium was treated with 25 µg/mL cefotaxime for 2 h (5). Subsequently, the cells were exposed to DMSO, Iso, CB1.11, or D66. We used LPM pH 7.0 for these experiments to increase outer membrane permeability enough to enable compound access to cells. After 2, 4, and 6 h, bacteria were plated for CFU enumeration, and biphasic kill curves were observed, consistent with enrichment for persister cells (4) (Fig. S5). As expected, after 6 h, DMSO and Iso had similar effects on bacterial survival. In contrast, CB1.11 reduced persister survival by 5-fold at 50 µM and 10-fold at 100 µM. D66 was less toxic to persister cells, with the 160 µM condition resulting in a sevenfold decrease in survival (Fig. 6A). Thus, the CB1.11 compound reduces the survival of cefotaxime-revealed persister cells in broth.

The high concentration of CB1.11 required to kill persister cells in broth may have reflected insufficient access of the compound to the inner membrane in LPM pH 7. Since macrophage phagosomes contain outer membrane-permeabilizing conditions beyond low magnesium and low phosphate, we evaluated the effect of the CB1.11 and D66 on persisters within cell culture macrophages. RAW264.7 macrophages accumulate few *S*. Typhimurium persisters at least in part because they produce little reactive nitrogen species, which promote the persister state (31). Therefore, iBMDMs were infected with *S*. Typhimurium and treated with 100 µg/mL cefotaxime to select for persisters (3, 5, 31–33). Cells were then exposed to DMSO, Iso, CB1.11, or D66. After 24 h, the iBMDMs were lysed and plated for CFU enumeration. Bacteria within DMSO- and Iso-treated cells survived similarly, as expected (Fig. 6B). However, persister populations declined twofold in D66-treated iBMDMs and sixfold in CB1.11-treated iBMDMs with only 20 µM of the compound. Furthermore, CB1.11-treated iBMDMs cleared *S*. Typhimurium in a dose-dependent manner. These data indicate that voltage-disrupting compounds such as CB1.11 and D66 have the potential to reduce persister populations.

## DISCUSSION

### PMB pre-screen enhances the efficiency of host-based drug library screens

The first step of our two-step screen was designed to use batch culture to enrich for compounds expected to be antibacterial in macrophages. While we do not know how many of the 16,799 library compounds are antimicrobial in macrophages, in a previous screen of the MayBridge HitFinder library, only 0.5% of 14,400 compounds were active in SAFIRE (12). This percentage compares well with the 0.48% of ChemBridge Library compounds we identified as active in SAFIRE. However, unlike the screen performed by Reens et al. (12), only 553 compounds required screening in macrophages to achieve this hit percentage, rather than the entire compound library. These data suggest that using a fast initial batch screen that incorporates PMB enriches for hit compounds with antimicrobial activity in a host cell infection environment.

Limitations of the screen included that neither screening step was automated. The use of a robot to aliquot reagents followed by automated absorbance readings and analysis would increase throughput rates and likely improve consistency. Automation would reduce the number of days needed for screening, potentially reducing the variation. For instance, we noted inconsistencies in control wells, which led to selection of some compounds as hits based on within-plate performance. Automation could also have made it feasible to screen all 16,799 library compounds with and without PMB. However, a counter-argument for this approach is that many compounds toxic to bacteria are also toxic to macrophages and are expected to be eliminated in the SAFIRE screen. We also found PMB to be variable in its potentiation of novobiocin; a different membrane permeabilization method could increase the consistency of control wells. For example, using a strain of *S*. Typhimurium with an *lptD4213* mutation or lacking an efflux pump subunit would allow greater compound access to the cell without the addition of a chemical. However, higher concentrations of compounds may need to be used in this case, as our *E. coli lptD4213* mutant and our efflux mutant strains had relatively high IC_50_ values compared to cells treated with PMB. Consequently, future screens may benefit from further optimization to balance screen efficiency with efficacy.

### Lipophilic compounds often emerge from SAFIRE screens

Overall, the hit compounds identified by the screening steps were distributed broadly across the chemical space of the ChemBridge library, whereas antibiotics generally clustered together (Fig. 1C). The original sources of the antibiotics are largely microorganisms, limiting the types of core structures to those that can be produced via naturally occurring, biosynthetic pathways (34, 35). In contrast, the ChemBridge library is highly diverse, covering a much larger swath of chemical spaces. Along these lines, eight of the ten antibiotics that plotted outside of the major antibiotic cluster are chemical derivatives of biologically synthesized products (Fig. S2; Table S3). Overall, these observations suggest there is a wide range of chemical spaces with potential anti-infective properties that are yet to be explored.

Among compounds identified by the two-step screen, the moderately lipophilic Class 1 compound CB1.11 (logP = 4.55) was particularly effective. This molecule was studied further because it was potent in the single-digit micromolar range against bacteria in macrophages and had relatively low toxicity. In standard broth cultures, CB1.11 was effective against gram-negative bacteria only under conditions that either permeabilized the outer membrane or compromised efflux pumps. While it is well-established that the phagosomal microenvironment weakens cell envelope barriers and increases small-molecule trafficking to the bacterial cell (22, 36), none of our efforts to permeabilize the outer membrane or inactivate efflux pumps reduced the IC_50_ to levels we observed in macrophages (Fig. 3B and D). However, the host cell produces other immune factors or insults that are not accounted for in our broth conditions and could synergize with CB1.11. For instance, in addition to nutrient starvation and acidic conditions, macrophage phagosomes accumulate combinations of cAMPs, reactive oxygen and nitrogen species, proteases, and copper that simultaneously attack bacterial cell envelopes (18, 37, 38). While cell culture-based screening methods are more resource-intensive, they nevertheless better mimic native infection conditions and may therefore be better able to identify chemicals with *in vivo* antimicrobial activity.

Beyond CB1.11, many of the hits from this screen and previous SAFIRE screens are somewhat lipophilic in nature, despite the fact that lipophilicity was not sufficient to predict activity in SAFIRE (Fig. S2). We speculate that because SAFIRE is conducted in macrophages, which load their phagosomes with defenses that permeate the bacterial outer membrane and occupy efflux pumps, there could be a bias toward lipophilic hit compounds. Previous broth screens with standard media would have likely missed detecting these lipophilic compounds because the gram-negative bacterial LPS layer limits their entry, and efflux pumps expel many molecules that do traverse the outer membrane (1). It is also apparent that compounds with higher logP values were identified with the initial PMB broth screen data, supporting the idea that membrane permeabilization is key for letting lipophilic compounds access gram-negative bacteria (Fig. S2B). Increased lipophilicity has also been a trend observed across three separate SAFIRE screens and has been noted in screens with *M. tuberculosis* (Fig. S2B) (39). *M. tuberculosis* has a distinct outer membrane that contains high levels of mycolic acids and is lipophilic in nature (40). Therefore, *M. tuberculosis* does not require membrane permeabilization for lipophilic compounds to enter the cell, a property that may account for the increase in effective lipophilic compounds observed in screens with this species. Gram-negative bacteria within macrophage phagosomes could be vulnerable to hydrophobic compounds much like *M. tuberculosis* in standard broth conditions. These findings suggest that lipophilic molecules contain properties that make them effective antimicrobials, particularly under host-like conditions.

### Lipophilic compounds can have limited host toxicity

Lipophilic compounds are generally considered risky to pursue as antimicrobials because of potential accumulation in host membranes that may result in toxicity (41, 42). For instance, host membranes could be at risk for insertion of CB1.11 because the compound did not appear to interact with specific classes of lipids during the MD simulations (Fig. S4). Despite these observations, CB1.11 demonstrated relatively little host cell toxicity, as revealed with MitoTracker Red staining and in LDH-release assays (Fig. S3). Mitochondrial and other host organelle membranes are likely protected from the activity of CB1.11 because they reside outside of phagosomes and thus are not attacked by innate immune factors. Along these lines, previous work found that compounds with a logP value below 6 minimally interfere with mitochondrial membranes (43). In the phagosome, the lipophilicity of CB1.11 could enable it to wedge effectively into the bacterial inner membrane and disrupt voltage. The CB1.11 isomer we identified, which is identical save for an ortho- rather than meta-substituted amide bond, is poorly able to insert into the inner membrane and lacks voltage-disrupting and bactericidal activity (Fig. 4B). That small differences in structure can significantly impact the activity of compounds has been observed previously in *M. tuberculosis* (44). While future screens could likely benefit from incorporating cell culture-based methods into their initial stages to avoid compounds with overt host cell toxicity, our data and those of other studies have shown that not all lipophilic small molecules are necessarily toxic to the host. Therefore, moderately lipophilic compounds warrant deeper investigation for antimicrobial development.

### Inner membrane voltage disruption is a key antimicrobial target

The lipophilic nature of CB1.11 led us to consider mechanisms of action related to PMF disruption in the inner membrane. We showed at both the population and single-cell levels that CB1.11 clearly disrupts the voltage gradient without concomitant rupture of the inner membrane nor of the inner membrane pH gradient (Fig. 5). Moreover, the voltage gradient is sufficiently disrupted to disperse a cell division protein, FtsZ, that relies upon this gradient to localize to the septum. Thus, disarming the inner membrane voltage of *S*. Typhimurium appears to disable the pathogen enough to let the macrophages kill and clear it. Other proteins essential for cell division, such as MinD or MreB, also rely on membrane voltage for correct localization (45). We hypothesize that CB1.11 induces inner membrane voltage disruption and consequently prevents essential cell division proteins from correctly assembling, likely making cell division impossible. Further, it is feasible that this voltage disruption is sufficient to weaken the PMF such that key inner membrane proteins, such as the ATPase, which is voltage-dependent (46), lose functionality. We cannot currently distinguish whether CB1.11 broadly inserts itself within the inner membrane to cause voltage disruption or if it also or instead interacts with inner membrane proteins. However, given that our screens and those conducted in *M. tuberculosis* have favored lipophilic hits that disrupt membrane voltage, we speculate that this arm of the PMF could be a good antibacterial target. Several FDA-approved drugs disrupt gram-positive bacterial PMF via voltage and pH disruption and also share the quality of being more lipophilic in nature (47). Feng et al. reasoned that the increased lipophilicity was an asset that allowed for inhibition of membrane proteins and collapsing the PMF as antimicrobials with multiple targets are also more difficult for bacteria to develop resistance against. While logP values of compounds tend to drop by ~1.0 between drug patents and launched drugs due to toxicity issues (48), by default, the SAFIRE screen selects for compounds with minimal host cell toxicity. Thus, CB1.11 and other hits from this screen are likely promising starting points for anti-infective development. Overall, our screen data add to the growing support behind the PMF being an important target for novel antimicrobials and that lipophilic compounds may be a reasonable scaffold for development of such PMF-disrupting compounds (11, 15, 16, 49, 50).

### Persisters are susceptible to CB1.11

The hostile macrophage environment results in the emergence of *S*. Typhimurium persister populations during infection (3). These persister cells do not actively proliferate within macrophages, making them a difficult population to treat with current antibiotic regimens, which typically target biosynthetic pathways of actively growing cells (51). In contrast, antimicrobials that target bacterial membranes are effective against non- and slow-growing populations and often disrupt inner membrane voltage, compromising the PMF (52–54). Thus, these membrane-targeting compounds are considered a viable means to potentially target persisters and other forms of recalcitrance in addition to actively growing pathogens. Since CB1.11 appears to disrupt inner membrane voltage and likely, by extension, PMF, it is perhaps not surprising that both in broth- and host-cell based persister assays, CB1.11 significantly reduced the persister population size. This suggests that CB1.11 is able to impact a population of bacteria that is not actively proliferating, which distinguishes it from antibiotics such as fluoroquinolones, cephalosporins, or aminoglycosides. We hypothesize that CB1.11 is effective against persisters because, though they are nongrowing when in a persister state, *S*. Typhimurium persisters are relatively metabolically active and require energy supplied by PMF to activate efflux pumps and DNA repair machinery, key aspects of their survival strategy (5). Selecting a ubiquitous target such as the inner membrane thus has a huge advantage as it is absolutely necessary for both growing and nongrowing populations. The efficacy of CB1.11 against persisters reiterates the utility for studying and adapting membrane-targeting, voltage-disrupting compounds since they target such a key facet of bacterial metabolism.

### Conclusions

Overall, this work offers insights to guide future screening efforts for compounds effective against gram-negative pathogens during infection. The small-molecule CB1.11 was effective at killing bacteria in broth and at enabling macrophages to kill actively growing bacteria and persister populations. Our evidence suggests that CB1.11 disrupts the voltage gradient of PMF, resulting in weakened bacteria that the host can kill and degrade. Despite lipophilicity, CB1.11 was minimally toxic to host cells at concentrations that enable macrophages to kill actively growing bacteria, indicating that voltage-targeting small molecules are a viable direction for antibiotic development.

## MATERIALS AND METHODS

### Strains and media

Bacterial strains and sources are listed (Table 1). MHB contained 2 g/L beef infusion solids, 1.5 g/L starch, and 17.5 g/L casein hydrolysate. LB contained 10 g/L tryptone, 5 g/L yeast extract, and 5 g/L NaCl. pH was adjusted to 7.0 using NaOH. LPM 7 (21, 22) contained 5 mM KCl, 7.5 mM (NH_4_)_2_SO_4_, 0.5 mM K_2_SO_4_, 10 mM glucose, 49 µM MgCl_2_, 337 µM PO_4_^-^, 0.05% casamino acids, and 80 mM Tris-HCl pH 7. LPM pH 5.5 contained the same ingredients as LPM 7.0 but was buffered using 80 mM MES at pH 5.5. Gram-positive strains were grown in TSB (BD, 211825).

**TABLE 1:** Bacterial strains and sources

Species	Strain	Antibiotic resistance	Source
S. Typhimurium	SL1344	STR	(55)
S. Typhimurium	SL1344 ∆*tolC*	STR	This study
S. Typhimurium	SL1344 *∆acrAB::kan*	STR, KAN	(12)
S. Typhimurium	SL1344 *sifB::*gfp	STR, KAN	(56)
*E. coli*	K12	–^*a*^	Coli Genetic Stock Center
*E. coli*	K12 *lptD*4213	–	Coli Genetic Stock Center
*E. coli*	K12 ∆*tolC*	KAN	Coli Genetic Stock Center
*S. aureus*	USA300	Methicillin	Horswill Lab; ATCC BAA-1717
*S. aureus*	FDA209	–	ATCC 6538
*S. aureus*	HG001	–	Horswill Lab; AH2183
*B. subtilis*	*B. subtilis*	–	ATCC 6633
*S. epidermidis*	*S. epidermidis*	–	Horswill Lab

^*a*^ "–” indicates no known antibiotic resistance.

### Construction of the *∆tolC* strain

The helper plasmid pAM053 (Table 2) , which carries a constitutively expressed CRISPR-Cas9 system (57) (58) and temperature-induced lambda red recombination system (42 °C for 15 min), was introduced into the SL1344 wild-type strain to generate the parent strain for genome editing. In plasmid pDJ6, Cas9 NGG (*AAATTGCTCCCCATCCTTAT*C**GG**) (Table 3) is designed to guide the Cas9 enzyme to create a double-stranded break within the *tolC-*coding region. The plasmid pDJ6 also contains a donor DNA template, where homologous sequences flanking the downstream and upstream of the *tolC* region without *tolC* CDS are supplied. pDJ6 was electroporated into SL1344. Successfully mutated strains were confirmed and sent for whole-genome sequencing.

**TABLE 2:** Plasmids used

Plasmid_Name	Source
pDJ6	This study
pAM053	(57)

**TABLE 3:** Primers used

Names	Sequences	Use
Primer_75	CATATGCGGCAATACGAATCTGTTCGACC	pDJ6 construction, *tolC* upstream
Primer_76	CATTCCTTGTTGTGAAGCAGTATTTAGC	pDJ6 construction, *tolC* upstream
Primer_85	GCTTCACAACAAGGAATGCATTGATAAGTTATTCGCTGGCGC	pDJ6 construction, *tolC* downstream
Primer_86	TAGTATTATACCTAGGACTGAGCTAGCTGTCAACGCAGTACGTTCGGCCT	pDJ6 construction, *tolC* downstream
Primer_87	TAGGTATAATACTAGT*AAATTGCTCCCCATCCTTAT*GTTTTAGAGCTAGAAATAGCAAGT	pDJ6 backbone
Primer_88	GAACAGATTCGTATTGCCGCATATGATCCAGCATATGCGGTGTGAAATAC	pDJ6 backbone

### ChemBridge chemical screen

ChemBridge Combi-PremiumSet Library compounds (16,799; quality-checked by mass spectrometry; ChemBridge, San Diego, CA USA) at 10 mM in DMSO (Sigma-Aldrich, St Louis, MI, USA) were screened in the first step as follows. Overnight cultures of *S*. Typhimurium SL1344 grown in MHB were diluted 1:100 in MHB with PMB (Sigma-Aldrich) at final concentrations of 0.75 or 1.0 µg/mL, as indicated (Table S1). We had previously established that the PMB concentrations used (0.75 or 1.0 µg/mL in MHB) permeabilize the *S*. Typhimurium outer membrane but not the inner membrane (14). Cultures were distributed into 96-well plates containing 50 µM of the compound, grown for 18 h with aeration at 37°C. OD_600_ was measured on an H1 Synergy plate reader (Illumina, San Diego, CA USA). Due to plate-to-plate variability, we sorted data from each plate individually and selected compounds with the lowest ODs to move forward, yielding 553 hits from the first step.

Hit compounds from the first step of the screen were retested for PMB-dependent growth inhibition with 0.5 µg/mL of PMB and 25 µM of compound. Hits were then verified using the SAFIRE assay, as described in 12. Briefly, RAW 264.7 macrophages (American Type Tissue Collection, Manassas, VA USA) were seeded at 5 × 10^4^ macrophages in 100 µL of complete DMEM per well in black glass-bottomed plates. The plates were incubated at 37°C with 5% CO_2_. The next day, overnight bacterial cultures of SL1344 containing a chromosomal *sifB*::gfp reporter grown in LB were diluted in PBS to achieve a final MOI of 30. Gentamicin was added 45 min post-infection to a final concentration of 40 µg/mL. After 2 h of infection, 25 µM of the hit ChemBridge compounds was added. At 17.5 h after infection, 100 nM MitoTracker Red CMXRos (Sigma-Aldrich) was added. Cells were then fixed 18 h after infection with a final concentration of 4% paraformaldehyde and incubated for 15 min. The cells were then washed with PBS and stained with 1 µM DAPI and stored in 90% glycerol in PBS until imaging.

Samples were imaged on a semiautomated confocal laser scanning CellVoyager CV1000 microscope with a 20×, 0.75 NA objective (Yokogawa & Olympus, Tokoyo, Japan), and bacterial accumulation via GFP fluorescence was quantified. Macrophages were defined by DAPI and MitoTracker Red dyes that stain for DNA and macrophage vitality based on mitochondrial voltage, respectively. To quantify bacterial accumulation, the number of GFP-positive pixels per macrophage was divided by the total number of pixels per macrophage and then averaged across all macrophages in the field (59). SL1344 CFU enumeration was performed as described above but with cells seeded in tissue culture plates. At 18 h, macrophages were lysed with 0.1% Triton X-100 and diluted for CFU enumeration on LB plates.

### tSNE cheminformatic analysis

tSNE cheminformatic analysis used the Python RDKit package. ChemBridge provided the molecule structures for the screening library. Canonical antibiotic structures were determined from their SMILES (see Table S2—ChemBridge 20K compounds), which were gathered from PubChem. Hydrogens were added to the molecule before calculating molecule coordinates using the *EmbedMolecule* function in RDKit. Hydrogens were removed before converting the molecule to its SMILE. MACCS keys (60) were generated from the SMILEs, and then the Tanimoto pairwise distance matrix was computed. tSNE (61) embedding was run with a PCA initialization and perplexity of 50.

### Principal component analysis

PCA for the ChemBridge library was performed on a curated set of physiochemical properties, including the following: molecular weight, logP, number of aliphatic rings, number of aromatic rings, fraction of sp³-hybridized carbon atoms, polar surface area, and number of hetero-atoms. Python package RDKit was used to compute molecular features from the ChemBridge-provided molecular structures. The features were Z-score-normalized using the sklearn python package prior to PCA. PCA was computed using the scikit-learn implementation.

### MIC assays

Overnight cultures of bacteria were grown in LB. The cultures were diluted 1:100 and then plated on a 96-well plate to which a dilution series of CB1.11 was added. If the strain was being tested in LPM 7, the overnight cultures were done in LB and then diluted 1:100 into LPM 7. After 18 h, OD_600_ was measured in a Biotek Synergy H1 plate reader (Illumina).

### Novobiocin assay

Overnight cultures of SL1344 were grown in LB and diluted 1:100 in a 96-well plate. Wells were treated with DMSO, 100 uM CB1.11, 0.5 µg/mL PMB, or no treatment. Each of these conditions was repeated with and without the presence of 4 µg/mL novobiocin (Sigma-Aldrich). After 18 h, the OD_600_ was measured using a Biotek Synergy H1 plate reader (Illumina). Biological triplicates were performed, and the means of each condition were analyzed by a two-way ANOVA and Tukey’s post test.

### Batch viability assay

Overnight cultures of SL1344 ∆*tolC* were diluted 1:100 and grown to mid-log phase (OD_600_ ~ 0.3) in LB. CB1.11 or Iso was added at the stated concentrations along with a DMSO control. Samples were collected at 0, 15, 30, 60, and 120 min. At each time point, an aliquot was taken to measure OD_600_ in a Biotek Synergy H1 plate reader (Illumina), and another was diluted for CFU enumeration via geometric viability assay in LB (62). CFU counts were performed the next day. Biological triplicates were performed.

### Molecular dynamics simulations

MD simulations were initialized using the Multicomponent Assembler module of CHARMM-GUI (27, 28). The thickness of the water above and below the membrane, as well as the membrane itself, was initially set to a thickness of 50 Å with a box length of 80 Å. The total system volume was 9.6e^5^ Å^3. The compounds were initialized to 10 Å above the membrane. The lipid composition for a gram-negative bacterial membrane was based on previous work (63). The system charges were neutralized with KCl. The simulation force field was CHARMM36m, and the system temperature was set to 37°C. The simulations were run using GROMACS (64, 65) using GROMACS files generated by CHARMM-GUI. The system was minimized for 5,000 steps before six rounds of equilibration using v-rescale and c-rescale temperature and pressure coupling, respectively (Fig. S4B). All other parameters were left as default. Production simulations were run for 20 ns with 2 fs step sizes and with Nose-Hoover and Parrinello-Rahman temperature and pressure coupling, respectively. Simulations were visualized using VMD.

### Membrane voltage DiSC_3_(5) assay

Overnight cultures of SL1344 were grown in LB broth and then subcultured into LPM 7 until they grew to mid-log phase (OD_600_ ~0.3). The potentiometric probe, DiSC_3_(5) was added to the culture to a final concentration of 2 µM and incubated for 15 min. The culture was then divided into a black 96-well plate, and an initial read was conducted at 650 excitation/680 emission with a read height of 7.25 mm in a Biotek Synergy H1 plate reader (Illumina). After this initial read, CB1.11, Iso, D66, or DMSO was added, and the plate was placed back into the plate reader. Reads were then taken continuously for 40 min. Biological triplicates were performed.

### FtsZ microscopy

Overnight cultures of SL1344 Δ*tolC* carrying GFP-FtsZ were diluted 1:100 in LPM pH 5.5 and grown to mid-log (OD600 0.25) and treated with either 2% DMSO, 100 µM CCCP, 90 µM D66, or indicated concentration of CB1.11 for 15 min or 30 min. Live cells were harvested with a 1 s spin in a mini-centrifuge and dripped on 1% LPM agarose pad for imaging. Cells were imaged on a Yokogawa Cell Voyager CV1000 Confocal Scanner System with a 100 ×/0.6NA objective by an investigator blinded to treatment conditions. Line scans of individual bacterial cells were performed using FIJI line scan analysis. Since the bacteria were slightly of different lengths, the bacterial length was normalized to 1 and then placed into 15 bins for size. Averages were then calculated based on the observations per each distance bin. Three biological replicates with 20 bacterial cells per condition were performed.

### PI assay

Overnight cultures of SL1344 ∆*tolC* were grown in LB. They were then subcultured into LPM 7 until mid-log phase (OD_600_ ~0.3). At time 0, compounds were added. DMSO was added at the same volume as the compound additions. 0.01% SDS was used as a positive control for inner membrane disruption. Samples were taken at 0, 15, 30, and 45 min. At each time point, an aliquot of sample was incubated with 10 µg/mL PI for 5 min. The sample was washed once with PBS and then loaded onto a black 96-well plate and read at 535 excitation/617 emission in a Biotek Synergy H1 plate reader (Illumina).

### Cytosolic pH measurements

Cytosolic pH was determined using the dye BCECF, AM. Overnight cultures of SL1344 were grown in LB broth and then subcultured into LPM 7 and grown until mid-log phase. BCECF, AM (10 µM) and 400 µM EDTA were added to the cultures and then incubated at 37°C for 1 h. EDTA was added to weaken the outer membrane so that BCECF, AM could enter the cell. Samples were transfered into a black 96-well plate, and an initial read was taken. The compounds were then added to the plate. DMSO served as a negative control, and 50 µM of the protonophore CCCP was used as a positive control. The plate was returned to the plate reader and then was continuously read at (excitation/emission) 490/535 and 440/535 for 35 min. Technical and biological triplicates were performed for each condition. Cytosolic pH measurements were determined by subtracting the ratio of emission intensity when the dye is excited at 490 nm vs 440 nm from the pK_a_ of BCECF, AM (6.97) with the following equation: pH = 6.97 – log_10_(490 emission/440 emission).

### Persister assays

We adapted the protocol for broth persister assays from Michaux et al. 2021 (66). Overnight cultures of SL1344 were grown in LB and then subcultured into LPM 7. After 2 h of outgrowth, 25 µg/mL cefotaxime was added to each condition. Post 2 h, a dilution series of CB1.11 or D66 was added; this was considered the 0 h time point. Samples were collected for CFU enumeration via geometric viability assay (GVA) (62); at 0, 2, 4, and 6 h in order to confirm the typical persister biphasic curve. Samples for GVA were incubated overnight at 37°C and counted the following day. The mean relative CFUs/mL calculated for each condition at 6 h was compared to the DMSO control using a Dunnett’s test. Three biological replicates were done for each condition.

For the iBMDM infection, we followed the protocol for studying antibiotic persistence *in vivo* from Michaux et al. 2021 (66) (section 3.2). iBMDMs were seeded at 1 × 10^6^ iBMDMs/well in 6-well tissue culture-treated plates and incubated overnight at 37°C with 5% CO_2_. The infection medium consisted of RPMI 1640 with 10% FBS and 2 mM glutamine. On the day of the infection, 45 µL of overnight culture of SL1344 grown in LB was opsonized in 170 µL infection medium and 20 µL of mouse serum for 20 min. Six-hundred microliters of infection medium was added to the opsonized bacteria, and then iBMDMs were infected with 30 µL of this opsonized mixture per well to obtain a final MOI of approximately 10. Plates were centrifuged for 5 min at 110 *g* and then incubated for 30 min. The media was then aspirated off and replaced with prewarmed infection medium containing 100 µg/mL cefotaxime and compound. The infection went for 24 h before being lysed with 0.1% Triton X-100 and then plated on LB plates for CFU enumeration the next day.

## Data Availability

Supplemental figures, tables containing the screened compounds and their chemical properties, and molecular dynamics simulations are available at https://github.com/c-asamoto/Asamoto-et-al-2025-SI.

## SUPPLEMENTAL MATERIAL

Supplemental material spectrum.03217-25-s0001.pdf Fig. S1 to S5; Supplemental table captions.

Table S1 spectrum.03217-25-s0002.xlsx List of the ChemBridge compounds screened during each stepof our screening process.

Table S2 spectrum.03217-25-s0003.xlsx Chemical properties of the compounds used to analyze thecompounds during the tSNE analysis.

Table S3 spectrum.03217-25-s0004.xlsx List of in-use antibiotics that grouped outside of the main in-use antibiotics cluster seen in Fig. 1C.

ASM does not own the copyrights to Supplemental Material that may be linked to, or accessed through, an article. The authors have granted ASM a non-exclusive, world-wide license to publish the Supplemental Material files. Please contact the corresponding author directly for reuse.
