# Supplementary material for: A two-step screen identifies a small molecule that disrupts membrane voltage and is effective against growing and persister gram-negative bacteria
Source: Microbiol Spectr. 2025 Dec 31;14(2):e03217-25. doi: 10.1128/spectrum.03217-25 (PMC12889115; doi:10.1128/spectrum.03217-25)
Supplement: Supplemental material — Fig. S1 to S5; Supplemental table captions. [file spectrum.03217-25-s0001.pdf]

Supplemental Figures and Table Captions for:

A Two-Step Screen Identifies a Small Molecule that Disrupts Membrane Voltage and is Effective Against Growing and Persister Gram-Negative Bacteria

Ciara K. Asamoto[a], Calvin A. Ewing[a], Christian T. Meyer[a1], Samual C. Allgood[a2], Matthew JG. Eldridge[b], Donald Evans[a3], Toni A. Nagy[a4], Grace L. Christensen[a5], Amy L. Crooks[a], Daqing Jiang[a], Sophie Helaine[b], Corrella S. Detweiler[a#]

[a]Department of Molecular, Cell, and Developmental Biology, University of Colorado Boulder, Boulder, CO, USA

[b]Department of Microbiology, Harvard University, Cambridge, MA, USA

[#]Address correspondence to Corrella S. Detweiler, detweile@colorado.edu

[1]Present address: Pharmacy and Pharmaceutical Sciences, St. Jude Children's Research Hospital

[2]Present address: Eurofins, Lancaster, PA, USA

[3]Pressent address: Institute for Behavioral Genetics, University of Colorado Boulder

[4]Present address: Department of Biochemistry, University of Colorado Boulder

[5]Present address: Department of Immunology and Infectious Disease, Cornell University

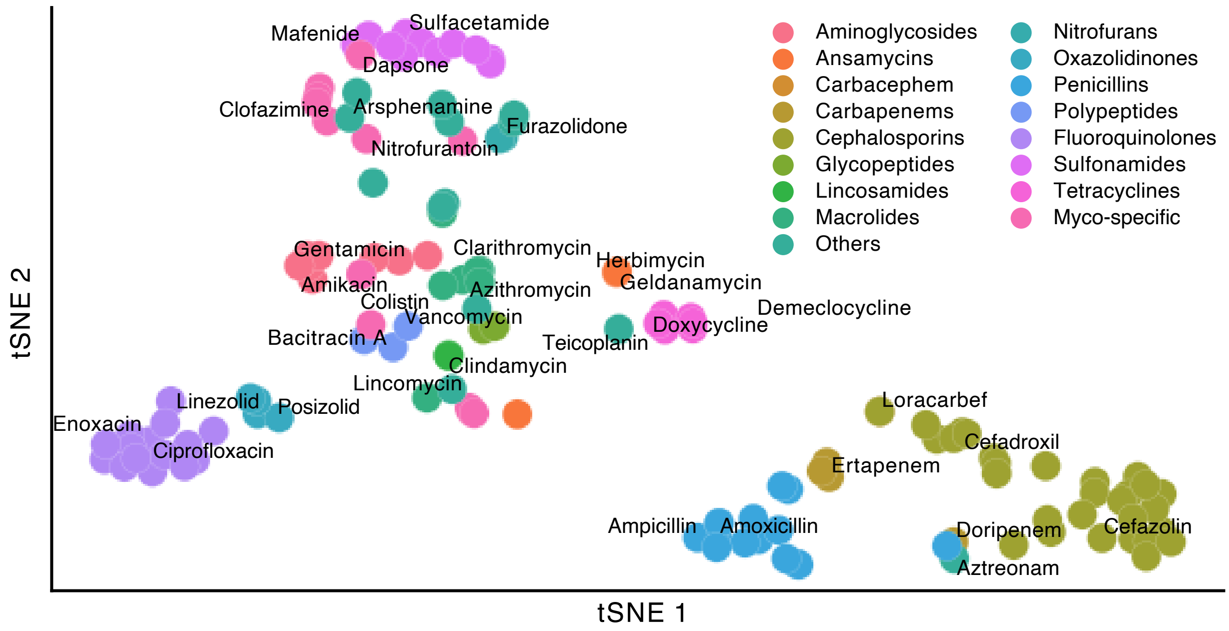

Figure S1. tSNE clustering of antibiotics independent of the ChemBridge library. Antibiotic classes are colored as shown in the key and two representatives of each family are annotated. Clustering is based on MACCS fingerprints and distances were calculated using Tanimoto similarity.

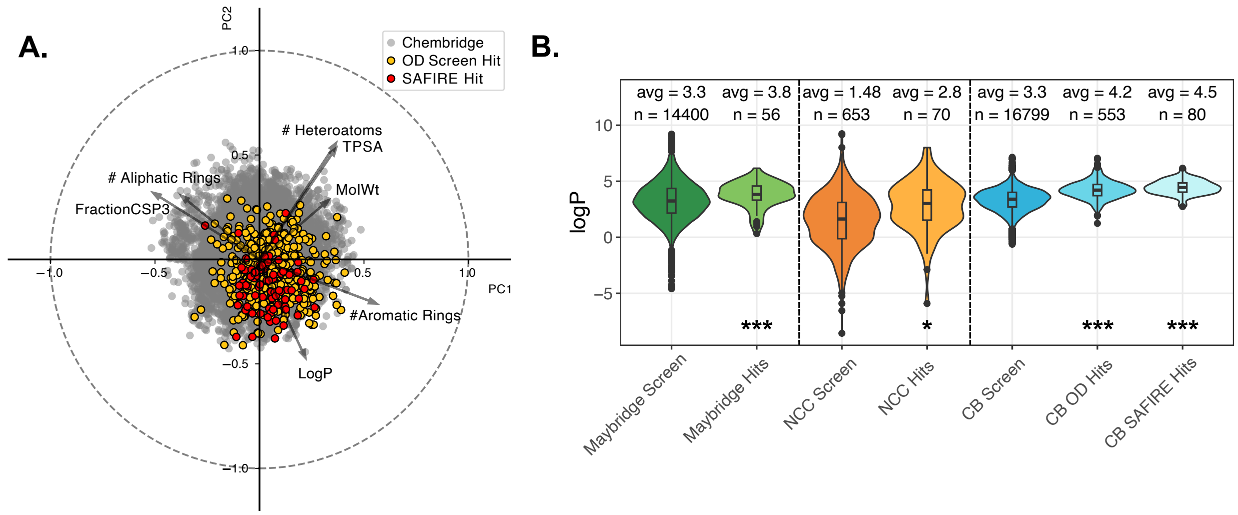

Figure S2. Slight enrichment of logP values found in this and previous SAFIRE screens.

A) PCA of the 16,799 Chembridge Combiset library compounds was performed on the physiochemical properties depicted in the plot. Molecular features were computed with RDKit and Z-score normalized with sklearn python package prior to PCA. Implemenation of PCA was performed with scikit-learn.

B) Mean and range of calculated logP values of compounds from the present study and two previous SAFIRE screens. Maybridge Hitfinder v11 Library (Maybridge; Reens et al. 2018), National Institutes of Health National Clinical Collection (NCC; Nagy et al. 2020), and Chembridge Combiset Library (CB; this study). LogP values were calculated with RDkit. The number of compounds in each library and set of hit compounds is annotated (n). The violin plot is scaled by width. The boxplots span the interquartile range with the horizontal line indicating the median logP value and outlier data plotted as points. A two-sample t-test was performed on the logP values from the hit compounds versus the parent library for each screen. $P < 0.05$, 0.005, 0.0005 denoted by *, **, and ***, respectively.

A.

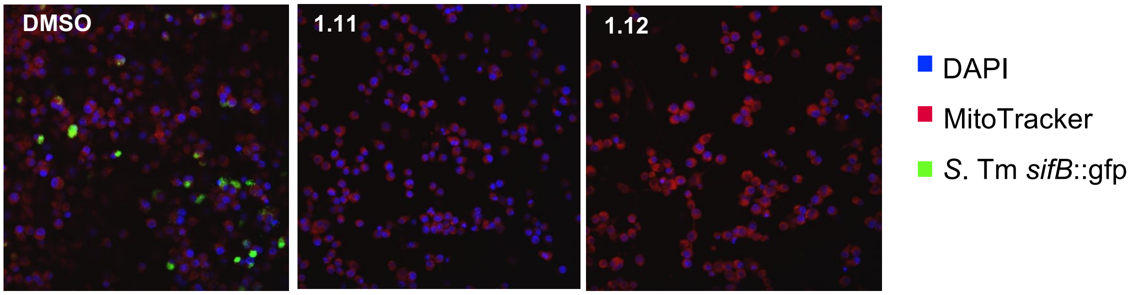

B.

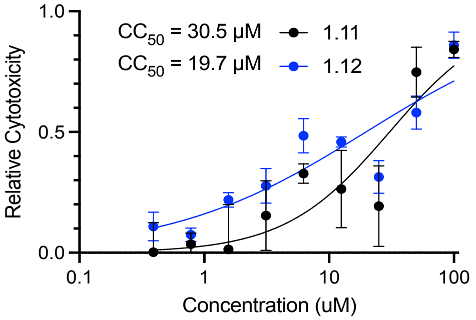

C.

Compound	CC_{50}	IC_{50}	Selectivity Index
1.11	30.5	3.9	7.8
1.12	19.7	2.5	7.9

Figure S3. Measures of host cell toxicity for compounds CB1.11 and CB1.12.
A) Representative images from the SAFIRE assay, in which RAW264.7 macrophage-like cells were treated with 25 µM of compound 2 hours after infection, incubated with MitoTracker Red after 17.5 hours, and fixed, stained with DAPI, and imaged after 18 hours of infection.
B) The lactate dehydrogenase (LDH) membrane integrity assay performed with RAW 264.7 cells at 16 hours after compound treatment. The key denotes half maximal cell cytotoxicity (CC_{50}) toxicity. Data are normalized to DMSO controls. Mean +/- SEM of three biological replicates.
C) Comparison of CC_{50}, IC_{50}, and selectivity index values for compounds CB1.11 and CB1.12.

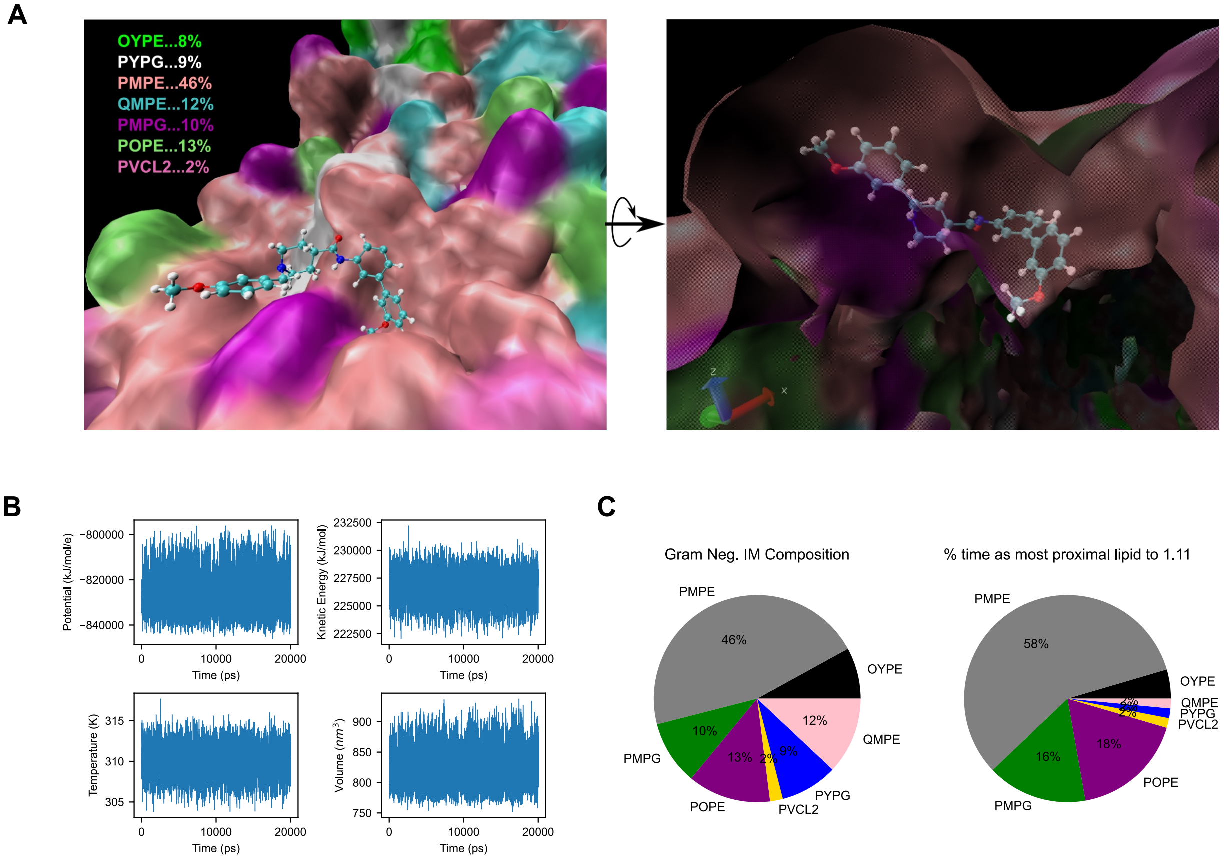

Figure S4. MD simulation, membrane energetics and system properties, and comparison of membrane lipid composition and compound proximity.

A) Model of CB1.11 inserting into the bacterial inner membrane.

B) Stable energetics and system properties for MD simulations. Time trace of potential and kinetic energy, temperature, and volume over 20 ns MD simulation post system minimization and equilibration. Simulations available in the GitHub repository associated with this paper.

C) (Left) Lipid composition of Gram-negative bacteria. (Right) Percent time lipid group is most proximal to CB1.11 during MD simulation. PMPE (plasma membrane phosphatidylethanolamine), PMPG (plasma membrane phosphatidylglycerol) and POPE (1-palmitoyl-2-oleoylphosphatidylethanolamine)

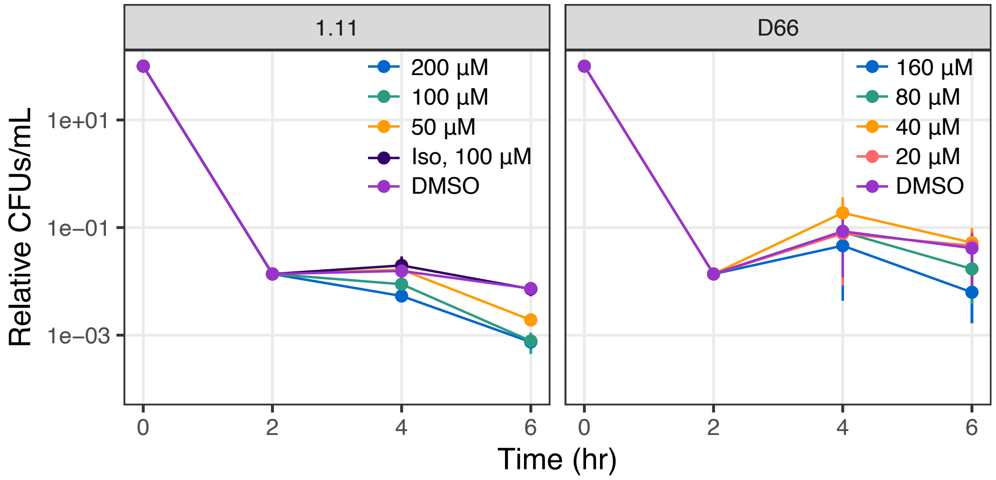

Figure S5. Biphasic kill curve for the broth persister assays.
S. Typhimurium was treated with 25 µg/mL cefotaxime at T0 to enrich for persisters. After two hours of cefotaxime treatment, DMSO, Iso, 1.11 or D66 was added. At each timepoint, samples for CFU enumeration were taken to assess *S*. Typhimurium viability. Each replicate was normalized to the T0 timepoint. Mean +/- SEM of three biological replicates.

Supplemental Table descriptions:

Table S1: List of the ChemBridge compounds screened during each step of our screening process. Contains compound ID, OD600 and/or percent inhibition in SAFIRE, and the chemical information for our top 80 hits.

Table S2: Chemical properties of the compounds used to analyze the compounds during the tSNE analysis.

Table S3: Derivations of the in-use antibiotics that clustered outside of the main in-use antibiotic cluster in Fig. 1C.

Supplemental references:

Nagy, Toni A., Amy L. Crooks, Joaquin L. J. Quintana, and Corrella S. Detweiler. 2020. "Clofazimine Reduces the Survival of Salmonella Enterica in Macrophages and Mice." *ACS Infectious Diseases* 6 (5): 1238–49. https://doi.org/10.1021/acsinfecdis.0c00023.

Reens, Abigail L., Amy L. Crooks, Chih-Chia Su, et al. 2018. "A Cell-Based Infection Assay Identifies Efflux Pump Modulators That Reduce Bacterial Intracellular Load." *PLoS Pathogens* 14 (6): e1007115. https://doi.org/10.1371/journal.ppat.1007115.
